# Serum-free media for the growth of primary bovine myoblasts

**DOI:** 10.1007/s10616-019-00361-y

**Published:** 2019-12-28

**Authors:** A. M. Kolkmann, M. J. Post, M. A. M. Rutjens, A. L. M. van Essen, P. Moutsatsou

**Affiliations:** Dept. Physiology, Maastricht University, 6229 ER Maastricht, The Netherlands

**Keywords:** Myoblasts, Xeno-free media, Proliferation

## Abstract

The demand for meat is expected to exceed production capacity by livestock in the coming decennia. Therefore, cultured beef might be a viable alternative to traditional livestock-derived beef. One of the problems however is the sustainability of cultured beef through the use of fetal bovine serum. We aimed to identify a serum-free medium or a serum-replacement that is as effective as the current method used for culturing bovine myoblasts. Cells were harvested from a female Blanc Bleu Belge cow and myoblasts were subsequently isolated. Cells were cultured in either Advanced DMEM containing 20% FBS and 10% HS or one of the chemically-defined, serum-free media for 6 days. MTS was used as a measure of cell proliferation at day 1, 4 or 6 and microscopic pictures were taken to assess cell morphology. FBM™, TesR™ and Essential 8™ are commercially available xeno-free media developed for human PSCs and fibroblasts, with the highest potential to sustain bovine myoblast proliferation. Of the supplements tested, XenoFree™ and a custom-prepared growth factor mix failed to stimulate cell proliferation. LipoGro™ stimulated cell proliferation in some cases but also changed the phenotype of myoblasts to an adipocyte-like phenotype. We conclude that serum-free media stimulate exponential cell expansion, albeit not to the extent of the current growth medium containing up to 30% serum. Further research is needed to investigate whether prolonged cell culture or an adaptation period could further increase cell proliferation.

## Introduction

Large scale production of primary mammalian cells is crucial to the success of cell therapy and tissue engineering for medical applications, but even more so for upcoming biotechnological solutions for commodities such as meat and leather. Production methods should be sustainable and therefore cannot rely on serum or other animal-derived materials that are non-replicative and therefore of limited supply (Post 2014). The use of serum is equally undesirable from animal welfare and regulatory perspectives. The qualitative and quantitative effect of batch variation of animal sera on cell culture has been well documented. As a byproduct of the livestock beef industry, there is also high possibility of viral, bacterial and endotoxin contamination of the serum (Fang 2017; Gstraunthaler Gerhard 2013). Additionally, from a financial point of view, the FBS market is very dynamic, leading to continuous price fluctuations rendering it unsustainable for large scale production. Indicatively, the price of US FBS has increased by 300% in the past few years (RMBIO 2016). Price volatility may not be a major issue in supplies for biomedical research, but will be devastating for large scale cell production to produce for example, meat.

The transition to completely serum-free media or at least the reduction of FBS in the culture medium is therefore indispensable for large scale production of biotechnological products, and especially for cultured meat as based on extrapolation of population growth and increase in welfare, meat demand is expected to increase by up to 70% in the coming decennia, whereas the amount of meat production through livestock may remain stable (FAO 2011; Gstraunthaler 2003). Reducing the demand side—eating less meat—cannot be well controlled at a global scale. The vegetarian population in Western societies is still a small minority and the decrease in meat consumption in Western Europe is offset by an increase in consumption in emerging economies (Leitzmann 2014). These realizations have led us to explore the possibility of culturing meat from primary bovine myoblasts, through large scale cell culture and tissue engineering, starting with a proof of concept by our lab in 2013 (Post 2014).

In traditional bovine myoblast culture, 20% fetal bovine serum and 10% horse serum are added to Advanced DMEM. Fortunately, xeno-free media and serum-replacements have been produced for a wide variety of cell types (Laitinen et al. 2016; Lindroos et al. 2009; Shetty et al. 2009). In this study, we aimed to identify commercial serum replacements in culturing primary bovine myoblasts.

A second problem with large-scale cell and tissue culture for food application is the routine addition of antibiotics and fungostatics to reduce the risk of infection. Ryu et al. have shown that PenStrep treatment of HepG2 cells during in vitro culture can significantly alter gene expression and regulation (Ryu et al. 2017) and Skubis et al. have shown that use of antibiotics has an impact on the phenotype of adipose derived stem cells, affecting both their proliferation and differentiation, but still little is known about the consequences of antibiotics-free culture of mammalian cells (Skubis et al. 2017). Therefore, the secondary aim of this study was to investigate the possibility and effects of myoblast cell culture in serum containing and serum-free serum medium without using antibiotics.

## Methods

### Harvesting and isolation of cells

A fresh post-mortem sample of skeletal muscle was taken from the *biceps femoris* of a Blanc Bleu Belge cow. After isolation, the muscle was cut into tiny pieces of about 8 mm^3^. These pieces were put into a Gentlemac C tube (Miltenyi Biotec, Germany) containing 400 units/ml collagenase 2 (Worthington Biochemical, USA) in Dulbecco’s Modified Eagle’s Medium (DMEM) (Life technologies, The Netherlands) containing 1% Penicillin/Streptomycin/Amphotericin-B (PSA, Lonza, Germany) to prevent bacterial and fungal growth. Next, tissue was further dissociated by the GentleMACS™ dissociator (Miltenyi Biotec, Germany) using the program m_heart_01.01. The content of the C tube was transferred to 10 ml centrifuge tubes and placed in a water bath (37°C) for 45 min and vortexed every 10 min. Finally, tubes were centrifuged (300×*g*, 10 min) to create a cell pellet.

### Start-up culture

The pellets were cultured after a pre-plating step (Sharifiaghdas et al. 2011), in 75 cm^2^ flasks at a seeding cell density of 1800 cells/cm^2^, coated with Matrigel™ (BD Biosciences, The Netherlands) diluted 1:200 in DMEM culture medium containing 1% PSA, until 85–90% confluency in Advanced DMEM (Life technologies, USA) containing 20% Fetal Bovine Serum (FBS, Life technologies, USA), 10% Horse Serum (HS, Life technologies, USA), 4 mM l-Glutamin (Lonza, Germany) and 1% PSA (growth medium: GM). Subsequently, medium was aspirated and the cells were washed with PBS. Next, cells were passaged by trypsinization with 0.05% trypsin-EDTA (Life technologies, USA) for 5–8 min at 37 °C, 5% CO_2_ in a humidified incubator and neutralization with TNS (Life technologies, USA). The resulting suspension was spun down at 460 g for 5 min at ambient temperature. After removal of the supernatant, cells were resuspended in freezing medium (Life technologies, USA) and stored in liquid nitrogen. Phenotype of the cells was confirmed by CD56 immunostaining and ability to form myotubes upon serum withdrawal (data not shown).

### Coating of culture plates and media experiments

96 well plates (Greiner Bio-one, The Netherlands) were coated with Matrigel™ diluted 1:200 in DMEM culture medium containing 1% PSA. The plates containing coating medium were incubated for 60 min at 37 °C, 5% CO_2_ in a humidified incubator. Plates were freshly prepared prior to experiments. After thawing, myoblasts were seeded at a density of 1800 cells/cm^2^ and cultured in several media (see below). GM, the medium used for culturing of myoblasts cells, served as benchmark for maximum growth and Advanced DMEM as minimum benchmark.

### Serum-free media

The serum free media that were tested for myoblast proliferation were: the Fibroblast basal medium with the FGM-CD SingleQuots Kit™ (FBM, Lonza, Germany), StemPro™ MSC SFM (StemPro™, Thermo Fisher Scientific, The Netherlands), the Essential 8™ Medium (Essential 8™, Life technologies, USA), the STEMmacs™ HSC Expansion Media XF (STEMmacs™, Miltenyi Biotec, The Netherlands), mTeSR1™ (mTesR1™, Stemcell Technologies, Canada), MesenCult™ ACF Culture Kit (Mesencult™, Stemcell Technologies, Canada) and TeSR™-E8™ (Stemcell Technologies, Canada).

### Media supplementation

Media that supported growth of myoblasts were additionally tested in the presence of two commercial serum-free additives, 5% LipoGro™ (Lipogro™, RMBIO, USA) and 6% XerumFree™ (XFS, TNC Bio, The Netherlands).

### Cell viability measurement

The number of viable cells in multi-well plates was estimated based on measurement of tetrazolium reduction as described in the following. MTS is a tetrazolium salt which is reduced by viable cells to generate formazan products that are soluble in cell culture medium. The conversion is carried out by NAD(P)H-dependent dehydrogenase enzymes. The number of sulfonated formazans delivered into the culture medium is quantified by measuring the absorbance at 490 nm and is directly proportional to the number of live cells in culture. The validity of the MTS assay to quantify cell number was validated by comparing visually acquired cell numbers obtained from a High Content Analyser (Becton Dickinson, Pathway 855) with MTS results in a subset of experiments. The two measurements correlated well, r^2^ = 0.982, n = 49.

Myoblasts were allowed to proliferate for 1, 4 or 6 days starting 24 h after thawing. Every other day 75% of the medium was refreshed. Prior to measurement, the medium was aspirated and completely replaced with fresh one to avoid inaccuracies due to evaporation and cells were incubated in a humidified incubator (37 °C, 5% CO_2_) for an additional 4 h. Next, 20 µl CellTiter 96^®^ AQueous One Solution Reagent (Promega, USA) was pipetted into 100 µl of culture medium. The reagent was incubated for 2 h in a humidified incubator (37 °C, 5% CO_2_) and measured directly with a 96-well plate reader (Viktor, PerkinElmer Life Sciences, USA) at a wavelength of 490 nm. Background correction was performed by measuring coated, medium filled, wells without cells. The resulting absorption was subtracted from the absorption obtained in the experimental conditions.

### Statistical data analysis

To correct for experiment-to-experiment variation, absorbance values of the MTS assay were standardized using the MTS value of growth medium on day 1. Conditions were tested for significant difference using one-way ANOVA with Tukey’s posthoc analysis or univariate analysis with two independent factors in SPSS statistics (Version 25 for Mac, IBM corp., USA). Unless otherwise stated, error bars indicate standard deviation.

## Results

**Fig. 1:**
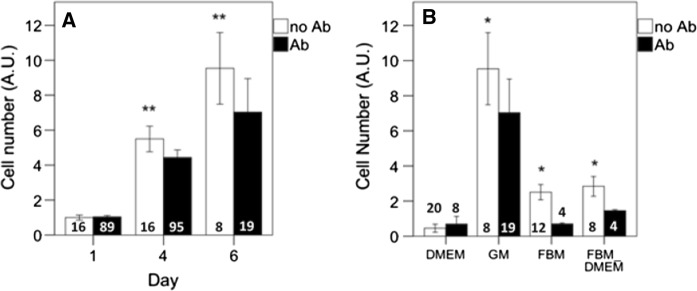
Effect of antibiotics supplement (Ab) on myoblast proliferation in standard growth medium (GM) over time (**a**) and in different serum-free media at day 6 of culture (**b**). The numbers at the base of the columns indicate number of observations. Asterisks indicate: *p < 0.05, **p < 0.01 indicate that mean values are significantly different from respective controls. Cell number was measured with MTS and expressed as arbitrary absorption values (AU). For medium abbreviations, see methods

To study the effect of antibiotics on cell proliferation over time, regular growth medium as well as several serum-free medium formulations were tested, in the presence or absence of 1% antibiotics (Penicillin/Streptomycin/Amphotericin B). In serum-containing growth medium, the antibiotic cocktail significantly reduced cell proliferation after 4 and 6 days of culture by 20% and 26% respectively (p < 0.01; Fig. 1a). Selected serum-free media were therefore also tested with or without the antibiotic cocktail (Fig. 1b). In the absence of serum, antibiotics had a similar growth inhibiting effect on bovine myoblasts as in growth medium containing serum with 62% reduction in FBM and 49% in FBM_DMEM (p < 0.05 for both). Our results are in direct agreement with Skubis et al. study on adipose derived MSCs, where a significant decrease in cell viability and increased mitochondrial oxidative activity was observed after 24 h, 48 h and 72 h of treatment of the cells with PSA (Skubis et al. 2017). They also found that amphotericin B caused a decrease of MSC markers at the mRNA level probably due to the impact of amphotericin B on mRNA stability.

In DMEM without serum, cells did not grow with a non-significant effect of number of culture days (univariate analysis: p = 0.59) and presence/absence of antibiotics (p = 0.96). The presence or absence of antibiotics did not affect the incidence of infections: throughout these experiments no infections occurred.

### Cell proliferation in serum-free media

**Fig. 2:**
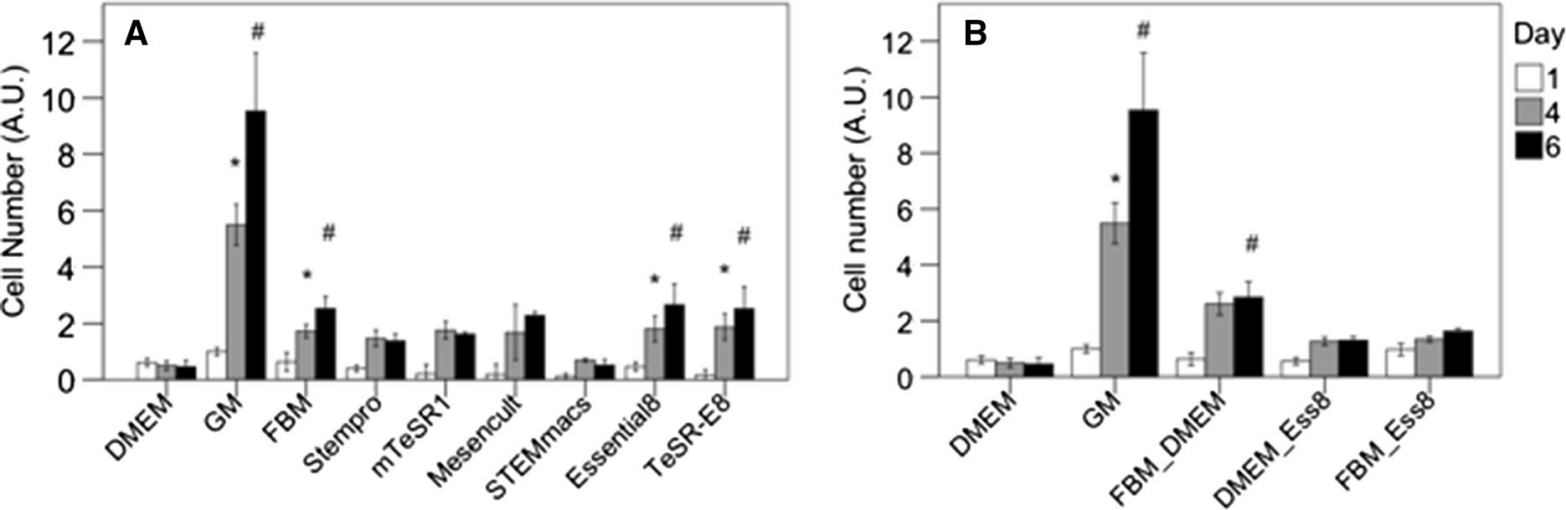
Growth of bovine myoblasts in serum-free media (**a**) and mixes of media (**b**) over time. Asterisks indicate significant cell growth from 1 to 4 and from 4 to 6 days. Pound sign shows significant higher cell number at day 6 versus DMEM only. Number of observations at 6 days: DMEM (n = 20), GM (n = 8), STEMmacs™ (n = 4), Stempro™ (n = 8), mTeSR1 (n = 4), Mesencult™ (n = 4), TeSR-Ess8 (n = 8), FBM (n = 12), Essential8™ (n = 16), FBM_DMEM (n = 8), DMEM_Ess8 (n = 4), FBM_Ess8 (n = 4). All serum-free media had lower growth than GM (p < 0.01). Cell number was measured with MTS and expressed as arbitrary absorption values (AU)

**Fig. 3:**
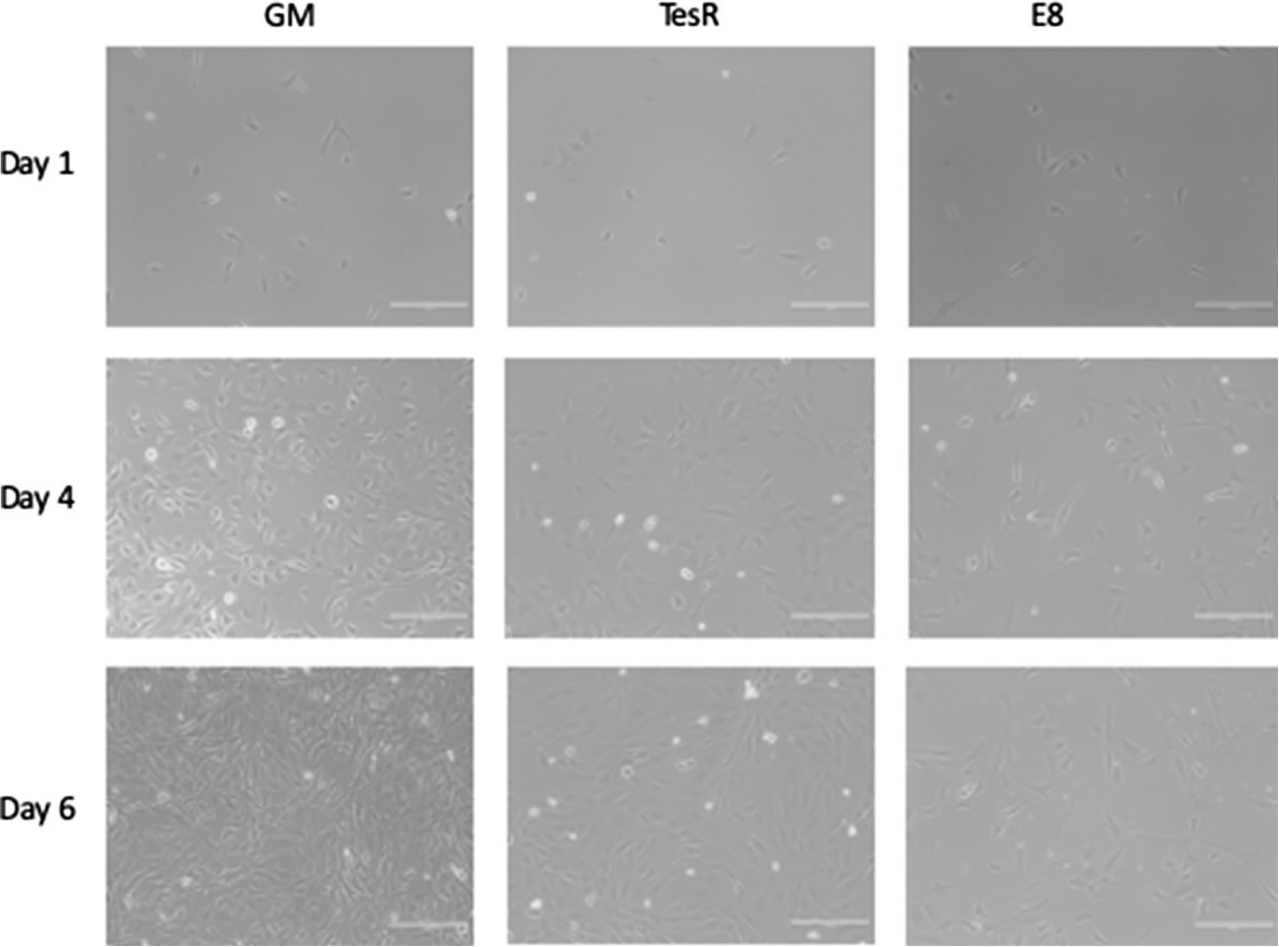
Phase contrast Photomicrographs of cell cultures at day 1, 4 and 6 with growth medium (GM), TesR™ and Essential8™ (E8). The morphology of the cells is not affected by serum-free media, but the numbers at day 4 and 6 are lower than for GM

Cells were cultured for 1, 4 or 6 days in growth medium or one of the chemically defined serum-free media (Fig. 2a). For all media tested, attachment of cells 5 h after plating the cells was unaffected (data not shown). Of the 7 commercially available serum-free media only FBM, Essential8™ and TESR-E8™ showed consistently increasing cell numbers over time, resulting in higher cell numbers after 6 days, than in culture medium without serum added (DMEM). No change in cell morphology was observed during this culture period (Fig. 3). Stempro™, mTESR1™ and Mesencult™ showed growth up to 4 days, but then stopped growing further. STEMmacs™ did not support growth of myoblasts. To further optimize serum-free medium for bovine myoblasts, media were mixed on a 50/50% basis. FBM was mixed with DMEM or Essential8™ and Essential8™ was mixed with DMEM. None of these mixes resulted in sustained growth over 6 days, although cell numbers at day 6 were higher in the FBM/DMEM mix than in DMEM without serum (Fig. 2b). A short-term effect of the medium was also observed at day 1. Cell numbers for serum-free media with the exception of FBM, were lower at day 1 when compared to GM, (p < 0.05 for all and p < 0.01 for STEMMacs™, Essential8™ and TeSR-E8).

### Addition of medium supplements

**Fig. 4:**
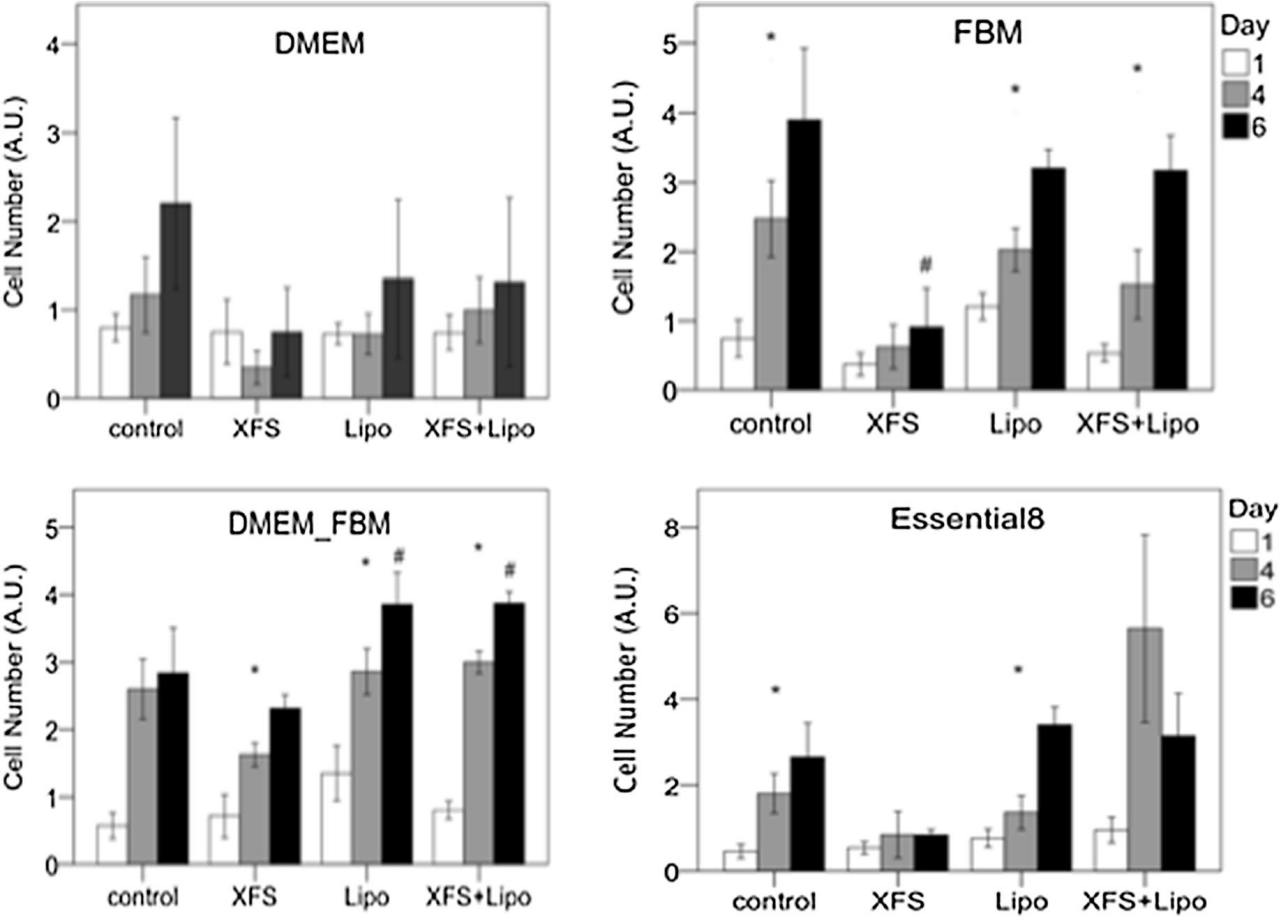
Myoblasts growth on serum-fee supplements XerumFree™ (XFS) and Lipogro™ (Lipo) added to serum-free media. Asterisks indicate significant cell growth from 1 to 4 and from 4 to 6 days. Pound sign shows significant higher cell number at day 6 versus DMEM only. Number of observations: FBM, n = 16, except for controls at day 1 (n = 8) and day 6 (n = 4); DMEM, control at day 6, n = 12, all others n ≥ 24; DMEM_FBM, n = 12, except for controls (n = 4); Essential8, day 6 Lipogro™, Xerumfree™, n = 4, all others n ≥ 16. Only in DMEM/FBM, Lipogro™ improved cell numbers at day 6; the combination with XerumFree™ did not have an additional effect. Cell number was measured with MTS and expressed as arbitrary absorption values (AU). For medium abbreviations, see methods

**Fig. 5:**
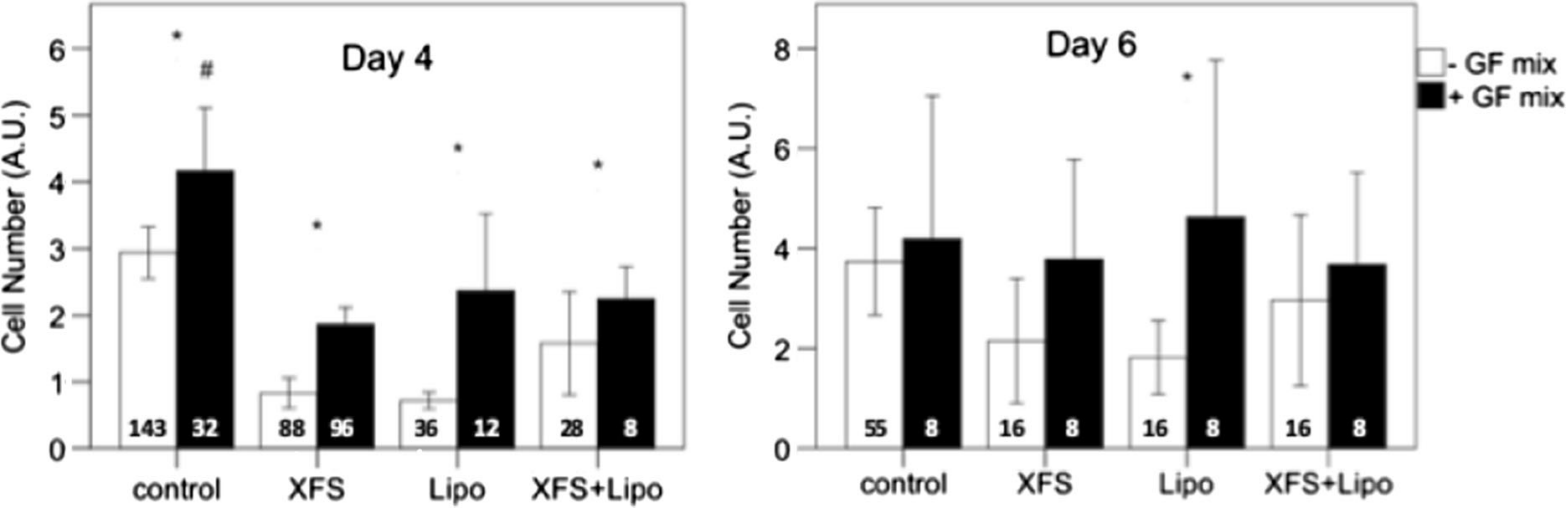
Effect of additional growth factors (GFmix = FGF-2 + insulin + EGF + IGF) on the effect of serum-fee media supplements on myoblast growth at day 4 and 6. The growth factor mix significantly increased cell numbers at day 4, although numbers were still lower in the presence of the supplements XerumFree™ and Lipogro™. At day 6, the growth factor mix increased cell numbers (F = 4.1, p = 0.044), although this effect was only significant for the Lipogro™ supplement. Asterisks indicate significant cell growth by the GFmix. Pound sign shows significant higher cell number at day 4 in the absence of supplements. The numbers at the base of the columns indicate number of observations. Cell number was measured with MTS and expressed as arbitrary absorption values (AU)

Supplements of horserum-free and fetal bovine serum-free media have been developed as an alternative to serum. Two such supplements, XerumFree™ and Lipogro™, were tested in DMEM, FBM, DMEM/FBM and Essential8™ (Fig. 4). In DMEM, neither of the supplements were effective in supporting myoblast growth. In FBM and the FBM/DMEM mixture, Lipogro™ allowed for myoblast growth but only in FBM/DMEM did this result in higher cell numbers after 6 days of culture when compared to the control (FBM or FBM/DMEM without the supplement). XerumFree™ was ineffective in promoting cell growth and especially in FBM, it resulted in an even lower cell number than the control condition. In Essential8, only Lipogro™ supported myoblast growth, but still without resulting in higher cell numbers at day 6, when compared to the control. The combination of DMEM/FBM and Lipogro™ seemed therefore the optimal serum-free condition for bovine myoblasts. As the supplements did not contain any growth factors, their addition to the cocktail was also tested. More specifically, 10 ng/ml FGF-2, 5 ng/ml EGF, 5 ng/ml IGF and 10 μg/ml insulin (Fig. 5) were added to the media. The growth factor mix resulted in better myoblast growth, regardless of the presence or absence of XerumFree™ or Lipogro™, although at day 6 only in the presence of Lipogro™ were the cell numbers higher than without the growth factor mix.

### Partial medium change

**Fig. 6:**
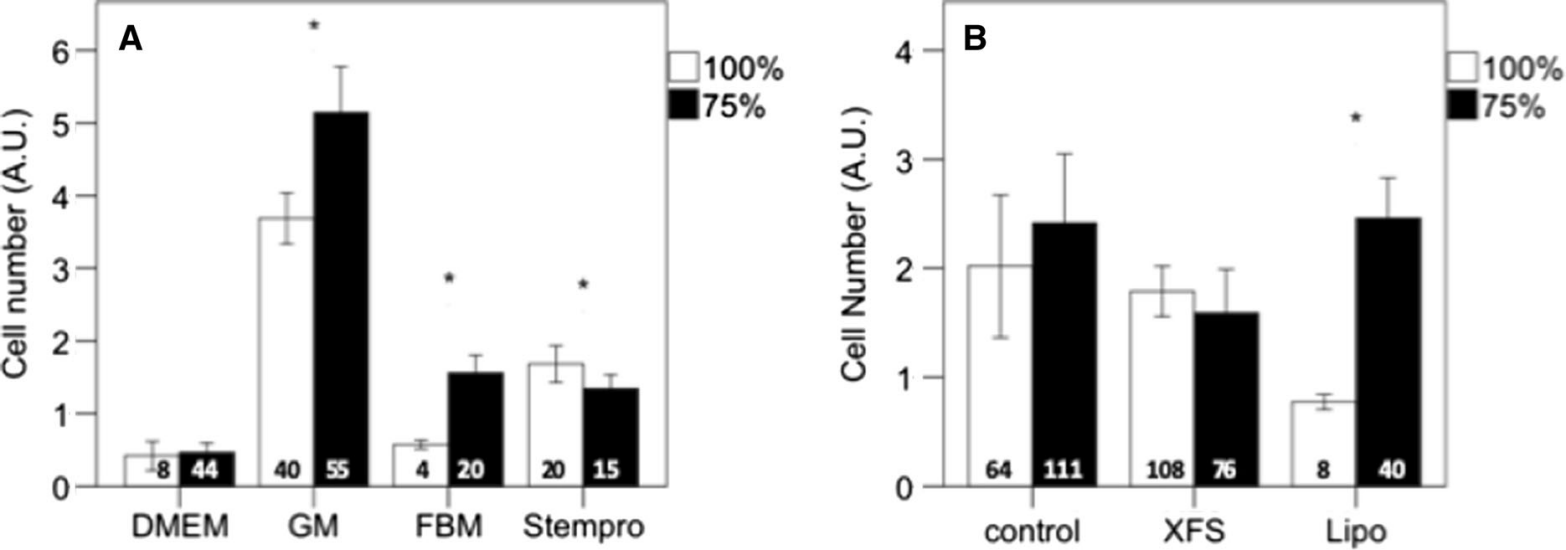
Effect of partial medium change on myoblast growth after 4 days of culture. In medium without supplements (**a**), partial (75%) medium change improved cell numbers over full medium change (100%). With supplements (**b**), the effect is less clear with only higher cell number after partial medium change in the Lipogro™ group. Asterisks indicate significant cell growth by the partial medium change. The numbers at the base of the columns indicate number of observations. Cell number was measured with MTS and expressed as arbitrary absorption values (AU)

Cells typically produce growth factors and it is assumed that they can thereby sustain their own growth. With serum-free media it might be better to only partially change the medium, leaving some of the produced growth factors in the culture. We tested the effect of changing only 75% of the medium. In the absence of supplements, 75% medium change improved cell growth, even for the GM. (n = 206, F = 4.2, p = 0.04, Fig. 6a). For Stempro™ however, cell numbers were lower with partial medium change than with a full medium change (n = 35, p = 0.009). For the supplements, the effect of partial medium change was less clear (n = 254, F = 3.9, p = 0.05, Fig. 6b), with higher cell numbers after partial medium change with Lipogro™.

### Morphology

**Fig. 7:**
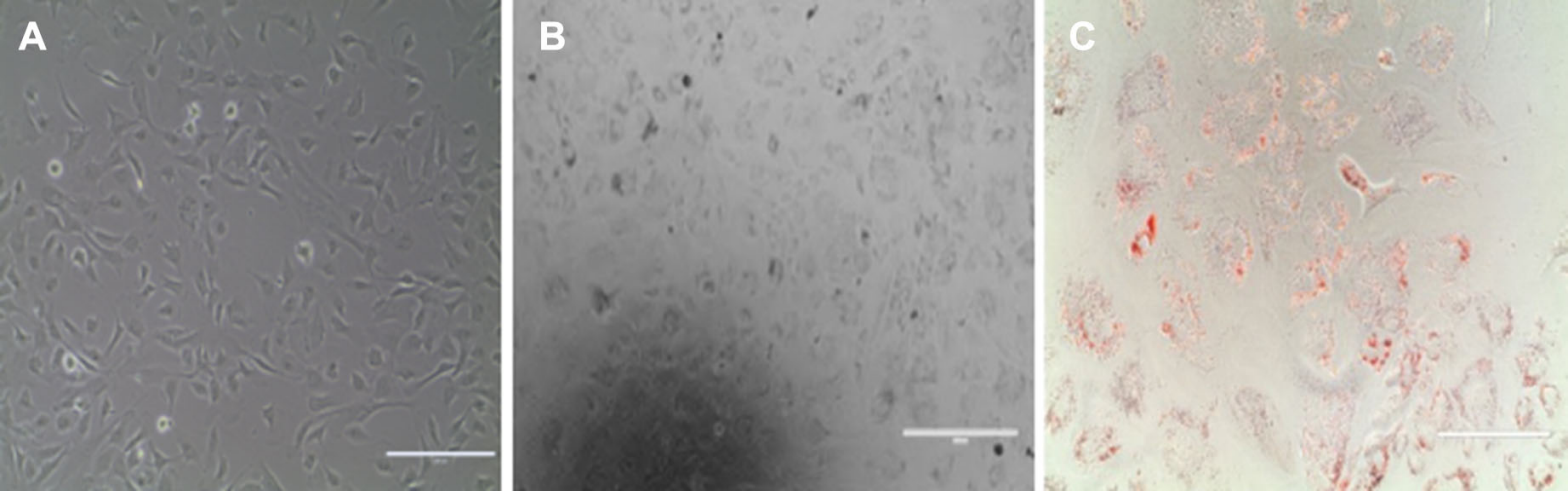
Photomicrographs of myoblasts cultured in DMEM (**a**), and DMEM supplemented with LipoGro™ (**b**, **c**). Oil-Red-O staining (**c**) confirmed adipogenic differentiation of myoblasts in the presence of LipoGro™. Bar = 200 μm

As Lipogro™ seemed to be a suitable supplement to replace serum in terms of supporting cell proliferation; we further investigated the phenotype of the cells (Fig. 7). Addition of Lipogro™ to the culture medium skewed the phenotype of myoblasts to an adipocyte-like structure with fat vacuoles inside.

## Discussion

In tissue engineering for medical applications, but especially for food applications, cell culture of a hitherto unimaginable scale will be developed. Even with current agricultural practices it is evident that these cultures cannot rely on serum as source of growth factors and essential trace components, a condition that has been called “Peak serum” (Bjare 1992; Brindley et al. 2012). In addition, regulatory requirements regarding the presence of serum traces in therapeutics or food, force elimination of serum from the production process (Hovatta et al. 2014). Also, there is increasing awareness of animal welfare aspects of drawing blood from unborn calves to produce serum (van der Valk et al. 2010). Numerous serum-free media and serum substitutes have been developed and tested for specific cells, starting with transformed cell lines, which are typically less serum dependent than primary cells. The existing commercially available serum-free formulations are also most frequently developed for human cell types because of the demand for serum-free and GMP compliant products from the cell therapy field.

For primary myoblasts, studies on serum-free medium are very limited. Allen et al have successfully grown and differentiated primary rat skeletal muscle satellite cells for 4 days in a custom-made serum-free medium comprising a mixture of DMEM and MCDB-104 as basal formulation supplemented with Deutsch fetuin, dexamethasone, insulin, transferrin, BSA-linoleic acid, selenium and FGF (Allen et al. 1985). The use of dexamethasone in a cultured meat process however is not desirable. Another serum-free medium has been developed by Shiozuka et al for chick satellite cells. FBS was replaced with a cocktail of transferrin, insulin, serum albumin, and FGF-2 as supplements to DMEM. The chick myoblast cells retained their ability to proliferate and differentiate. This serum-free medium also suppressed the proliferation of contaminating fibroblasts, but its efficiency in maintaining a long-term (> 4 days) culture of cells had not been identified (Shiozuka 2000).

In this study we tested 7 commercially available serum-free media and 3 supplements. FBM and Essential8™ consistently supported myoblast proliferation, although cell numbers did not reach the level of the high benchmark of 20% Fetal Bovine Serum and 10% Horse Serum. This suggests that the eventual number of doublings in these serum-free media will be lower than for serum-containing medium and that these media are still sub-optimal. However, when compared with culturing bovine myoblasts with 10% FBS, cell numbers are equivalent (data not shown) to that of FBM or Essential8™.

There is no gold standard for the isolation and culturing of bovine myoblasts (Motohashi et al. 2014; Pasut et al. 2013) and the conditions may thus be chosen to optimize cell numbers and therefore maximal number of doublings using aggressive growth media with high serum concentration. It remains to be seen if this is the proper strategy as the eventual goal is to create a maximum number of differentiated muscle fibers from a minimal sample size. In addition to delayed cell growth in the absence of serum, cell numbers at day 1 were also significantly lower in serum-free media with the possible exception of FBM (Fig. 2a). This can be due to either early cell death or reduced adhesion (Kim et al. 1992) to the coating with subsequent cell loss. Further studies are required to determine the cause of variation in cell numbers directly after seeding.

Serum is a highly complex solution containing proteins, growth factors, cytokines, cholesterol, free fatty acids and numerous other compounds that might influence cell attachment and growth (Bjare 1992). The eventual optimization of a serum-free medium will likely be the result of a systematic effort of many stakeholders to address the wealth of variables.

In addition to different basal media, we also tested available serum replacing supplements. The supplement XerumFree™ that was chosen did not really add anything to the effect of FBM and Essential8™ as basal media. Surprisingly, the addition of growth factors such as EGF and IGF-1 to these media that already contain FGF-2 and insulin, did not induce myoblast proliferation either. Each of these proteins has shown to induce myoblast proliferation or proliferation of a wide variety of cells including those of mesenchymal and epithelial origin and we expected to see an enhancing effect on myoblast growth. Higher concentrations or different mixtures might be tested, but the concentrations that we used are commonly used and typically effective in cell culture. Our experience with the supplement Lipogro™ that induced adipogenesis of bovine myoblasts is an illustrative example of cell numbers not being the only outcome that should be analyzed in testing culture conditions. Lipogro™ is a mixture of lipoproteins extracted from animal blood and is not a sustainable alternative to FBS. It was tested to study the effect of lipoproteins on cell growth and differentiation. Interestingly, the effect of Lipogro™ depended on the composition of the basal medium. Only in the combination of FBM with DMEM did Lipogro™ further support myoblast growth. Another interesting finding of this study was that Lipogro™ induced a change in phenotype of the myoblasts to a more adipocyte-like cell containing lipid droplets. Lipogro™ is a cholesterol concentrate and may therefore induce lipid-uptake in myoblasts. These intracellular lipids might lead to changes in metabolism and gene expression, leading to a differentiation towards adipocytes (Beloor et al. 2010). This property as well as the fact that Lipogro™ is a cholesterol concentrate derived from serum make that Lipogro™ is unsuitable for future use in meat production.

We considered the possibility that growing cells self-maintain or propagate further growth through the production of growth factors and other proteins. To test this, partial medium replacement that preserves 25% of these presumed cell products for the culture, was performed, by replacing only 75% of medium. This regimen did result in higher growth of myoblasts in some of the tested serum-free media, but also in serum-containing medium, suggesting that conditioned medium supports cell growth in a self-propagating manner. This also implies that partial medium replacement does not lead to enhanced nutrient depletion and accumulation of metabolic waste. Beneficial effects of partial medium change on cell growth was previously established for insect cells (Ikonomou et al. 2004). Changing the regimen of medium replacement thus turns out to be yet another factor to consider when optimizing cell culture for growth.

An encouraging finding is that antibiotics are not required in the culture of bovine myoblasts and that elimination of antibiotics resulted in higher cell growth. In none of the experiments did we observe contamination. Cell culture in the absence of antibiotics is an old practice and was published by Alexis Carrell (1924).

In summary, our data suggest that FBM, FBM/DMEM and Essential8™ have the potential to become as effective as the current serum-containing medium when it comes to cell proliferation. However, further investigation is needed to enhance cell attachment and cell survival during the first days of culture and to find a combination of growth stimulating factors that enhances cell growth to a level that is comparable to the serum containing medium. For cultured meat applications where the goal is to produce huge cell numbers, prolongation of the culturing period should be evaluated to check for the maximum growth potential of serum-free medium.
